# Base of Thumb Fractures: A Review of Anatomy, Classification, and Management

**DOI:** 10.7759/cureus.76729

**Published:** 2025-01-01

**Authors:** Zubair Younis, Muhammad A Hamid, Thomas Devasia, Muhammad Murtaza Khan, Faliq Abdullah, Rohit Singh, Adrian William Simons

**Affiliations:** 1 Orthopedics, The Royal Wolverhampton National Health Service (NHS) Trust, Wolverhampton, GBR; 2 Orthopedic Surgery, University Hospitals Birmingham, Birmingham, GBR; 3 Trauma and Orthopedics, The Royal Wolverhampton National Health Service (NHS) Trust, Wolverhampton, GBR; 4 Trauma and Orthopedics, The Princess Royal Hospital, Telford, GBR; 5 Trauma and Orthopedics, Royal Shrewsbury Hospital, Shrewsbury, GBR; 6 Orthopedics, Shrewsbury and Telford Hospitals National Health Service (NHS) Trust, Shrewsbury, GBR

**Keywords:** bennett fracture, intra-articular fractures, metacarpal fractures, rolando fracture, thumb base fractures

## Abstract

Fractures of the thumb metacarpal base are uncommon but significant due to their critical role in hand functionality and hand grip strength. These fractures exhibit diverse patterns, including extra-articular, Bennett, Rolando, and highly comminuted fractures, each with unique implications for management and outcomes. Each type presents unique challenges in achieving anatomical reduction, stability, and favorable long-term outcomes. This review explores the anatomy of the trapeziometacarpal joint, classification systems, clinical presentation, imaging techniques, and management strategies for these fractures. Stable extra-articular fractures often respond well to closed reduction and casting, while displaced intra-articular fractures generally require surgical intervention. Bennett fractures are typically treated using closed reduction and percutaneous pinning or open reduction and internal fixation. Rolando and comminuted fractures pose greater challenges due to their inherent instability and often necessitate advanced techniques such as locking plates, external fixation, or arthroscopic-assisted procedures. Achieving anatomical reduction is paramount to prevent complications such as joint incongruity, instability, and post-traumatic arthritis. Optimal treatment approaches depend on fracture patterns, stability, and patient-specific factors, with surgical techniques tailored to preserve thumb function and minimize long-term morbidity.

## Introduction and background

The base of the thumb metacarpal is susceptible to various types of fractures, often resulting from axial loading along the axis of a partially flexed thumb [1]. These fractures represent around 4% of all hand fractures, with a higher prevalence in men, and may be associated with fractures of adjacent structures such as the trapezium [2-4]. They account for 80% of all thumb fractures, with the most common pattern being extra-articular epibasal fractures.

Among intra-articular thumb base fractures, the eponymous patterns described by Bennett and Rolando dominate the literature [5,6]. Around 30% of thumb metacarpal base fractures are Bennett fractures, making them about four times more common than Rolando fractures [7]. Both are typically caused by axial forces applied to the thumb in flexion, resulting in intra-articular instability. Inadequate reduction of these fractures increases joint contact pressures, increasing the risk of early osteoarthritis. In severe cases, excessive apex dorsal angulation can cause MCP joint hyperextension deformities, highlighting the importance of anatomical reduction [8].

Extra-articular fractures, including those at the metaphyseal-diaphyseal junction (commonly termed epibasal fractures), are frequently encountered and generally more stable. Diagnosis of all thumb base fractures is best achieved through orthogonal radiographs, which help determine fracture patterns and stability.

Despite the thumb’s critical role - contributing nearly 50% of overall hand functionality - treatment strategies for thumb base fractures remain the subject of ongoing debate [9,10]. Historically conservative treatment was favored, but research indicates that relying solely on closed reduction and casting often leads to unfavorable results, particularly in cases of unstable fractures [11]. Displaced intra-articular fractures, including Bennett and Rolando fractures, typically require surgical fixation. Techniques range from skeletal traction and external fixation to internal fixation with wires, screws, or plates, as well as other osteosynthesis techniques such as percutaneous pinning, open screw fixation, and locking plates [12]. While these approaches aim to preserve thumb function and minimize secondary arthritis, arthroscopic and percutaneous techniques carry a risk of neurovascular injury and secondary displacement [13].

## Review

Anatomy

The trapeziometacarpal joint (TMJ) has a biconcave-convex shape, resembling two interlocking saddles [1]. This unique structure allows for both flexion-extension and abduction-adduction motions, providing a wide range of movement essential for thumb function. A volar beak prominence on the metacarpal articulates with a recess in the trapezium [1]. Although 16 ligaments of the TMJ are described, the primary stabilizers are considered to be the volar oblique ligament and the dorsal ligament complex [14]. Connecting the tuberosity of the trapezium to the volar-ulnar base of the first metacarpal, the volar beak ligament serves as a crucial anchor between these structures. The dorsoradial ligament spans from the dorsoradial tubercle of the trapezium to the dorsal base of the first metacarpal, providing further stabilization. The volar oblique ligament is commonly regarded as the "essential" stabilizer as it opposes the dorsoradial displacement of the metacarpal base [15]. Furthermore, a dynamic "screw-home torque" mechanism may contribute to the stability of the thumb base. However, some studies highlight its laxity and suggest that stability is primarily provided by the dorsal ligaments [16]. This mechanism involves compressing the volar beak into the recess of the trapezium during the final phase of opposition, with tension in the dorsal ligament complex generating articular compression (indicating that the volar oblique ligament does not serve as the pivot point) [16].

**Figure 1:**
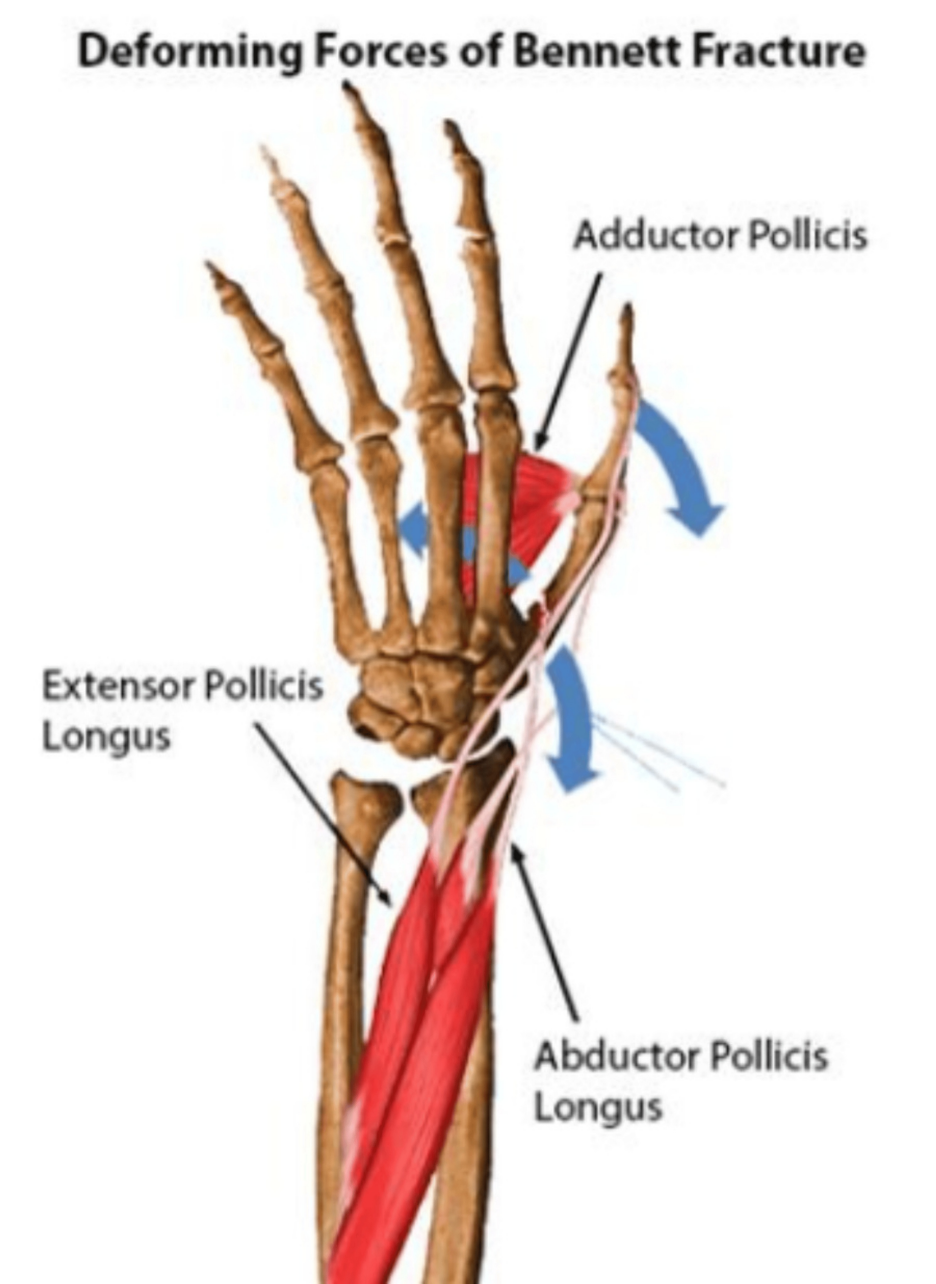
Deforming forces of Bennett fracture Source: Orthobullets. Reproduced with permission [18]

In Bennett and Rolando fractures, dorsoradial subluxation results in reduced metacarpal height, adduction deformity (varus alignment), and contracted first web space [17]. Three key muscles exert deforming forces at the base of the thumb, influencing its stability and motion. The abductor pollicis longus, innervated by the posterior interosseous nerve (PIN), applies proximal, dorsal, and radial forces on the distal shaft. In a similar manner, the extensor pollicis longus, generates force directed proximally, dorsally, and radially. The adductor pollicis, supplied by the ulnar nerve, exerts supination and adduction forces on the shaft fragment, assisting in thumb stability (Figure 1).

The carpometacarpal (CMC) joint has limited axial rotation, with an average flexion-extension range of 53° and an average abduction-adduction range of 42°. This complex interplay of bones, muscles, and ligaments enables the thumb’s versatility, which is essential for gripping and manipulating objects.

Classification

The fractures of the first metacarpal base can be classified into five distinct types based on the fracture pattern and involvement of the articular surface. Extra-articular oblique fractures feature an oblique fracture line that does not involve the joint surface, whereas extra-articular transverse fractures exhibit a purely transverse fracture line, also excluding the joint surface. Bennett fractures are intra-articular and characterized by a fracture with a palmar ulnar fragment. In contrast, Rolando fractures are intra-articular and present as complete intra-articular fractures with a Y- or T-shaped configuration. Finally, intra-articular comminuted fractures are defined by a severely comminuted, complete intra-articular fracture pattern. This classification serves as a valuable tool for understanding fracture types and guiding appropriate treatment strategies for first metacarpal injuries.

**Figure 2:**
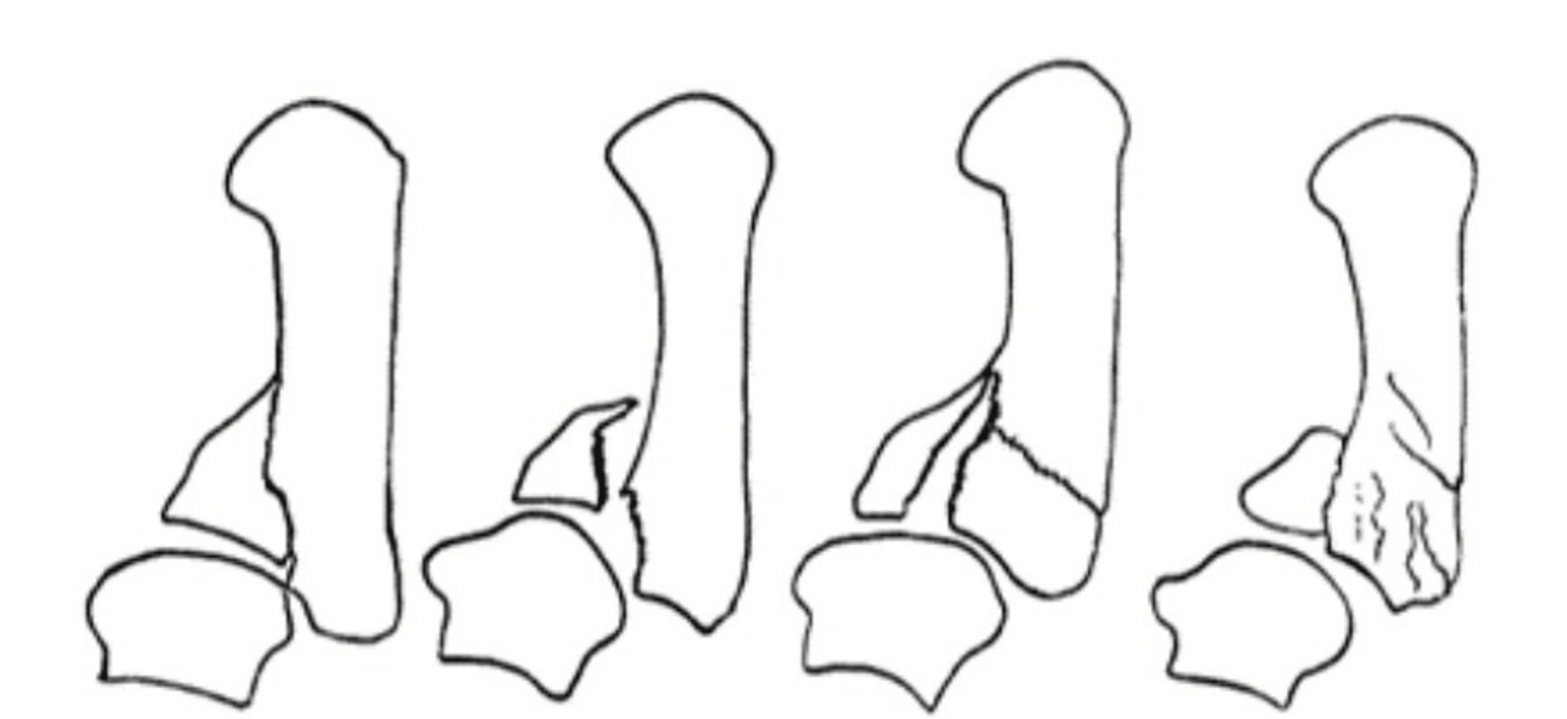
Gedda type 1 Bennett fracture Reproduced with permission [20]

**Figure 3:**
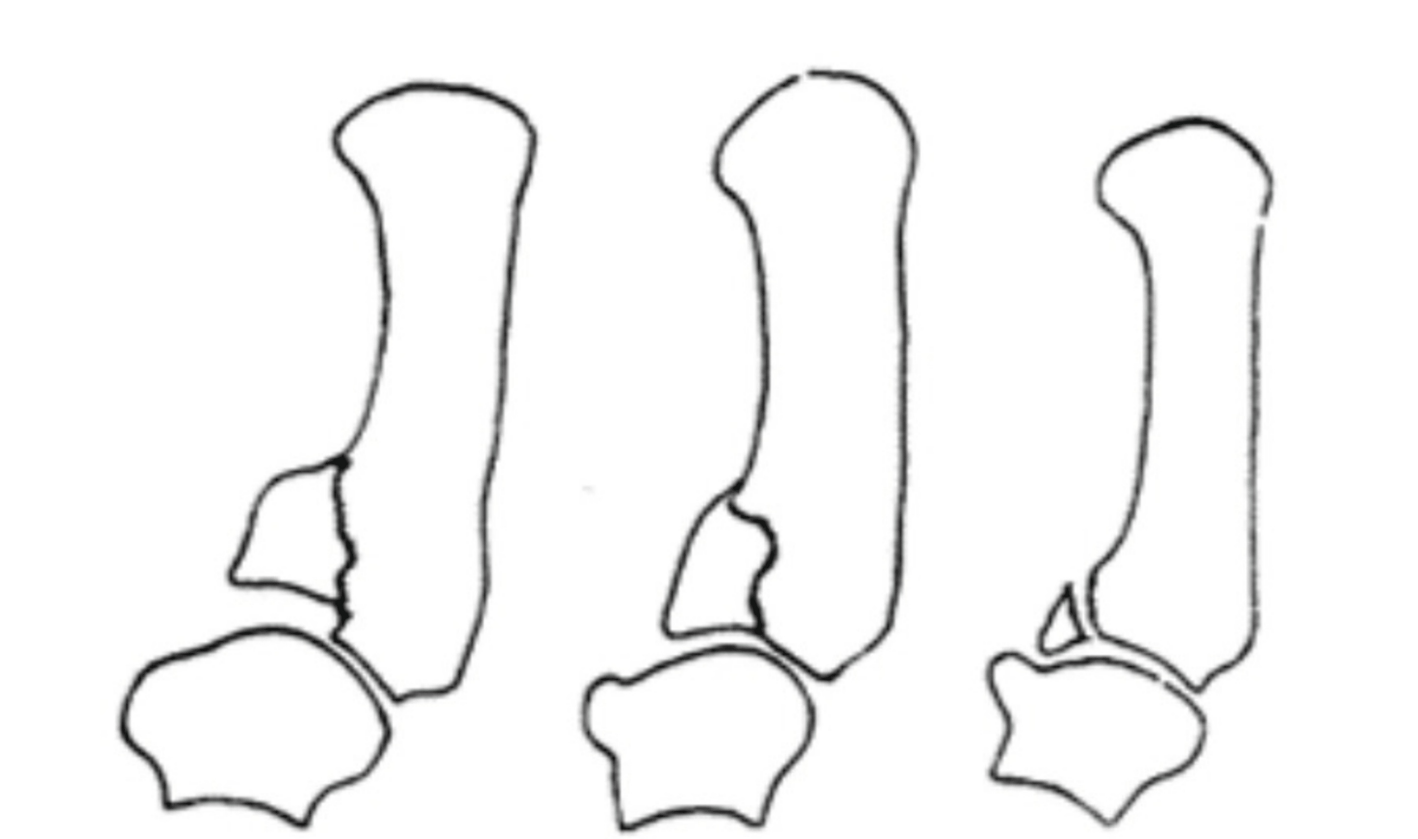
Gedda type 2 Bennett fracture Reproduced with permission [20]

**Figure 4:**
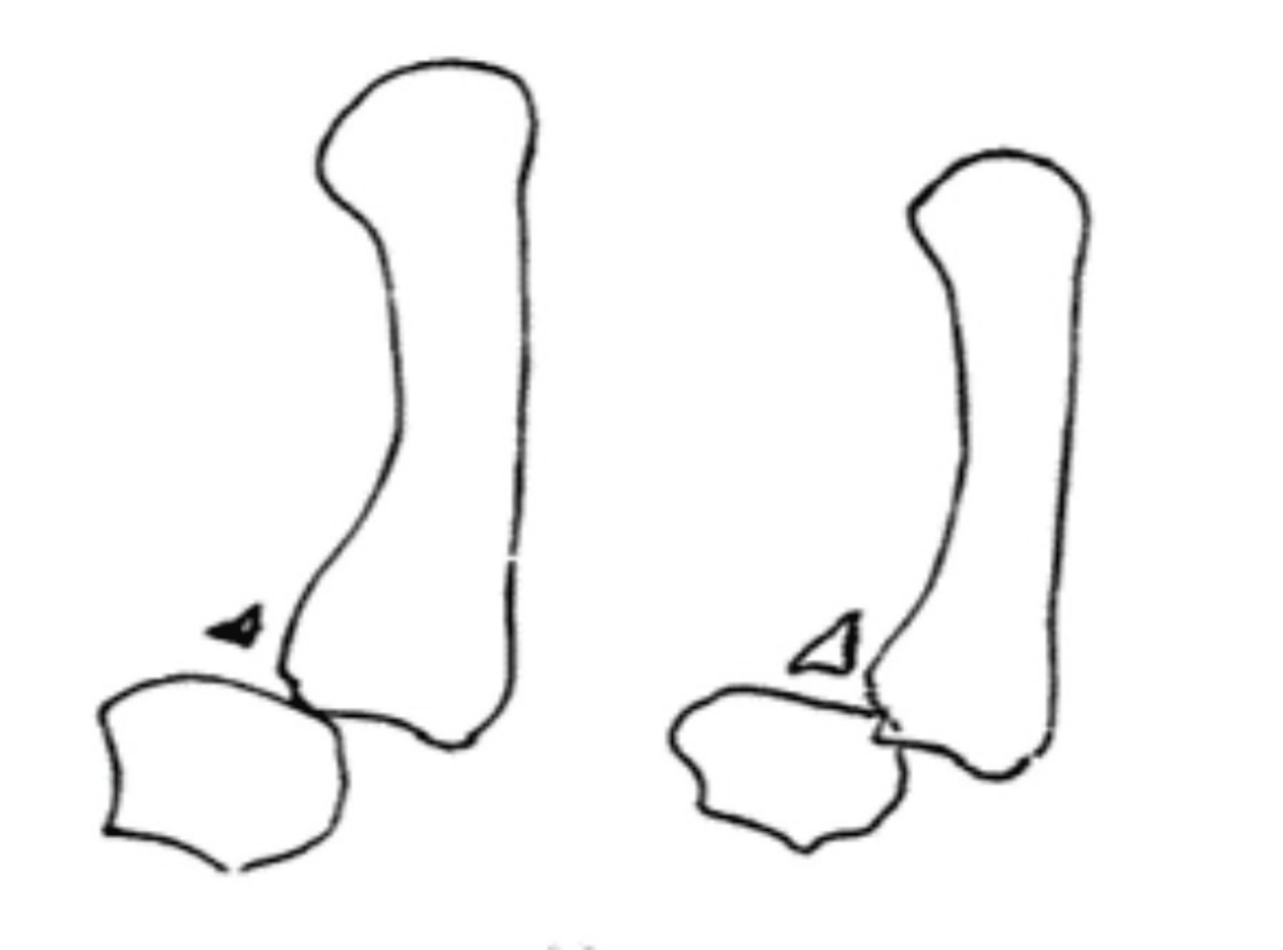
Gedda type 3 Bennett fracture Reproduced with permission [20]

Historically, Bennett fractures were further categorized by Gedda in 1952 into three distinct types [19]. The Gedda classification categorizes thumb metacarpal base fractures based on their characteristics and involvement of the TMJ. Type 1 fractures are intra-articular, often accompanied by subluxation of the metacarpal, with or without additional basal fractures. Type 2 fractures occur through the palmar tip of the metacarpal and are impacted but lack any associated dislocation or subluxation. In contrast, Type 3 fractures are characterized by small avulsion fragments with complete dislocation of the TMJ (Figures 2-4) [20].

Clinical presentation

Thumb metacarpal fractures commonly present following trauma, with acute pain localized to the base of the thumb. While not all fractures are symptomatic [5], thorough history and physical examination remain crucial for diagnosis. During physical examination, swelling and ecchymosis are often apparent upon inspection. Palpation of the CMC joint typically reveals tenderness, pinpointing the injury site. Crepitus may also be detected during attempted movement, further supporting the diagnosis. Attempts at the range of motion frequently provoke pain, suggesting joint involvement and potential fracture instability.

Radiology

Radiographs are essential for identifying and classifying thumb base fractures. A true anteroposterior (AP) view, known as Robert's view, is obtained by hyperpronating the hand so that the dorsum of the thumb rests on the imaging cassette [21]. This view may be modified slightly to correct minor TMJ offsets [22]. The Gedda or Bett's view, which involves pronating the hand at 15-25° and angling the X-ray beam at 15°, is the recommended technique for lateral imaging. Additionally, an oblique view may provide another perspective, which can be particularly helpful for delineating fracture details. On AP radiographs, the metacarpal base appears convex, while it appears concave on lateral views [23].

Optional imaging techniques, such as traction radiographs, are valuable for understanding complex fracture patterns, especially in Rolando fractures or severely comminuted injuries [24]. CT scans may further aid in evaluating intricate fragment configurations and guiding surgical planning. Intraoperative fluoroscopy is commonly used to assess articular congruity and guide reduction and fixation, though it may lack the precision of direct radiographic or visual evaluation [25]. Occasionally, TMJ arthrography can complement fluoroscopy for additional detail [16].

Key findings on imaging guide both diagnosis and management. Bennett fractures typically show a small fragment at the base of the first metacarpal articulating with the trapezium, while Rolando fractures can be characterized by the "Y sign," represented by splitting of the metacarpal base into dorsal and volar fragments [18]. Treatment is recommended when extra-articular fractures have angulation greater than 30° or when Bennett fractures present with an articular step-off exceeding 1 mm. In Rolando fractures, the degree of comminution often determines the surgical approach. A 30° pronated view has been noted for its high sensitivity and specificity in evaluating these injuries, making it a critical component of imaging [26].

Management

Bennett Fractures

A Bennett fracture is defined as an intra-articular two-part fracture-subluxation of the thumb metacarpal base featuring a single volar beak fragment, first described by Edward Bennett in 1882 [5]. There are two fragments: the larger fragment, comprising the bulk of the first metacarpal, undergoes an initial dorso-radial subluxation of the TMJ driven by the abductor pollicis longus, followed by adduction under the influence of the medial thenar muscles, which narrows the first web space. The smaller, variably sized volar beak fragment, representing the anteromedial corner of the thumb metacarpal base, remains fixed and undisplaced due to its attachment to the trapezium via the volar oblique ligament.

The treatment options for Benett fractures are controversial with treatment options varying from conservative management with early mobilization to open reduction and internal fixation (ORIF), encompassing various closed pinning techniques [27]. The initial treatment for these fractures begins with reduction, specifically focusing first on reducing the thumb metacarpal. Typically, the dislocation is reduced using external manipulation. Thumb abduction is initiated to correct the narrowed first web space and to relax the abductor pollicis longus. Then, using fluoroscopy, direct pressure is applied to the base of the thumb metacarpal within the anatomical snuffbox. The reduction is usually straightforward. Achieving a perfect reduction of this articular fracture is essential to prevent articular incongruence.

The second stage of treatment focuses on maintaining the reduction, with several methods available, ranging from conservative approaches like early mobilization to surgical options such as ORIF. However, there is no consensus on an optimal method, as each has limitations. High-quality articular reduction remains the primary goal, despite ongoing debate about the relationship between malunion and functional outcomes [28]. Conservative treatments, such as plaster immobilization, often fail to maintain a reduction in unstable injuries, and external fixation with continuous traction is uncomfortable and difficult to manage [29]. Surgical treatment is generally the most reliable for restoring joint congruence and stability.

Operative management of Bennett fractures has evolved since Lambotte first described the use of a pin to secure the volar beak fragment in 1913 [30]. Wagner later introduced retrograde trans-articular pinning with uniformly good outcomes reported for up to four years [31]. Bennett fracture-dislocations can be treated through percutaneous fixation, open surgery, or arthroscopy [32-34].

The minimally invasive technique of closed reduction and percutaneous pinning (CRPP) with trans-articular and/or intermetacarpal Kirschner (K)-wires has become increasingly popular, often making the pinning of the volar beak fragment unnecessary [35]. Stability in intermetacarpal wire constructs can be further enhanced by external connections [36]. Percutaneous osteosynthesis predominantly relies on pins, which are used for direct or indirect fixation, either crossing or avoiding the fracture site. These pins are favored for their ability to minimize the risk of extensor tendon adhesions. Among these techniques, the Iselin method, involving double intermetacarpal extra-articular pinning, is recognized as one of the most reliable approaches [35]. However, it is associated with complications such as secondary displacement, pseudarthrosis, malunion, post-traumatic arthritis, infections, reduced thumb mobility, and narrowing of the first web space. While CRPP carries risks like pin migration, infection, and post-traumatic arthritis, it remains the first-line treatment for displaced Bennett fractures due to its consistently favorable outcomes, provided the residual articular step-off is <1 mm [36].

ORIF offers several advantages, including the removal of ligament or capsular interposition, precise anatomical reduction under direct visualization, and stable fixation using screws or supplemental K-wires [9,19,35]. The surgical approach can be dorsal or palmar, with fixation options such as plates or screws, including simple screws, compression screws, and cannulated screws [33,35]. Despite these advantages, ORIF is associated with risks such as tendon adhesions, nerve injury, hardware failure, and a reduction loss rate of up to 30% [9,23,35].

Arthroscopic techniques offer significant advantages, including reduced soft tissue and vascular damage, improved articular visualization, and lower risks of tendon adhesions and bone necrosis [34]. These benefits stem from the minimally invasive nature of the procedure, which facilitates the removal of ligament or capsular interposition and allows direct visual control of joint surface reduction, potentially reducing the risk of post-traumatic arthrosis. Axial thumb traction further enhances arthroscopic setup and control over articular reduction quality.

However, arthroscopy has notable limitations, such as being technically demanding and requiring longer operative times. Traditionally, a single K-wire is used in these procedures, but it often fails to prevent secondary displacement due to its inherent instability. To address this issue, the use of double screw fixation has been proposed, offering improved stability and preventing rotational movement of small fragments [34]. Despite these advancements, challenges remain, including a steep learning curve, longer setup and tourniquet times, and increased financial costs. Additionally, percutaneous screw fixation may be limited by the inability to achieve fracture reduction through closed manipulation [37].

Given these limitations, current evidence supports CRPP as the first-line treatment for most displaced Bennett fractures due to its minimally invasive nature and consistently favorable outcomes. ORIF and arthroscopy are typically reserved for irreducible or complex fractures, where anatomical reduction cannot be achieved through less invasive methods [38]. Nevertheless, arthroscopic reduction and internal fixation remains a valuable option in selected cases, offering precise control over reduction quality and the potential for superior long-term outcomes.

Rolando Fractures

Rolando fractures are uncommon intra-articular Y- or T-shaped fractures of the thumb metacarpal base, characterized by an extra-articular transverse fracture separating the diaphysis from the epiphysis, with a vertical intra-articular line splitting the epiphysis into volar and dorsal fragments. Each fragment undergoes specific displacement due to muscular forces, often resulting in central joint depression and complex fracture patterns. These fractures are challenging to manage as closed reduction is typically unsuccessful, necessitating strategies focused on achieving anatomical reduction. While it is debated whether precise reduction prevents symptomatic osteoarthritis, no definitive correlation has been established between reduction quality and long-term outcomes. Each fragment exhibits a distinct pattern of displacement: the large distal diaphyseal fragment is pulled into adduction, narrowing the first web space due to the action of the medial thenar muscles; the lateral epiphyseal fragment is displaced upward and outward under the influence of the abductor pollicis longus; and the medial epiphyseal fragment remains stationary, anchored to the trapezium by the oblique posteromedial ligament [27].

The management of Rolando fractures primarily aims to achieve anatomical reduction, restoring the fractured bones to their original alignment. This is followed by stable fixation to enable early mobilization and preserve the range of motion of the TMJ, ultimately reducing pain [39]. However, the treatment of these fractures is challenging due to their inherent instability and comminuted patterns, often leading to complications such as loss of reduction, joint incongruity, and the potential development of osteoarthritis [10].

As a three-fragment articular fracture, a precise reduction is crucial to prevent articular incongruence, which is a known cause of arthritis [40]. Conservative management often fails to maintain reduction due to the fracture's complexity, as identified by Silvio Rolando, and cast immobilization frequently results in loss of reduction [6,41]. External manipulation is generally ineffective due to the fracture's complexity, and both conservative management and direct percutaneous osteosynthesis often produce suboptimal results. Moreover, studies, have shown that TMJ pinning not only fails to achieve satisfactory reduction but may also result in iatrogenic joint damage [42].

ORIF is preferred for large fragments, permitting anatomical reduction and stable fixation through lag screws and locking T-plates. These methods provide interfragmentary compression, facilitate early mobilization, and have demonstrated good functional outcomes [43]. External fixation remains a viable alternative for fractures involving small basal fragments or those associated with soft tissue injury, though outcomes vary, and complications such as pin site infections are common [44].

A recent study has introduced an innovative surgical technique for treating Rolando fractures, which are complex intra-articular fractures at the base of the thumb metacarpal [45]. This method involves implanting an endoprosthesis into the CMC joint of the thumb following injury to the base of the first phalanx. The primary goal is to achieve anatomical reduction and stable fixation, thereby facilitating early mobilization, restoring optimal range of motion, and reducing pain. This approach is particularly considered for young patients with multifragmentary fractures who are at high risk of degenerative disease in the CMC joint. However, it's important to note that this technique is not yet a standard treatment for these fractures, and more studies are needed to validate its efficacy and safety.

Comminuted Intra-articular Fractures

Highly comminuted intra-articular fractures, often regarded as severe variants of Rolando fractures, pose significant challenges due to fragment size, multiplicity, and associated soft tissue injuries [17]. The goals of treatment are to restore joint alignment, maintain the length of the metacarpal, and prevent the narrowing of the web space, though no consensus exists on the optimal surgical approach [17].

Closed reduction techniques, such as intermetacarpal pinning, preserve the first web space, while external fixation, relying on ligamentotaxis, aids in restoring anatomy [36,44]. Gelberman et al. reported successful reductions with outrigger thumb traction, though detailed outcomes were limited [44]. The method described by Buchler et al. involves combining external fixation with open reduction and limited internal fixation, demonstrating good functional outcomes despite some joint irregularities [46].

Adjunctive options like TMJ arthroscopy assist in reducing fractures and removing loose fragments [47,48]. Salvage procedures, including arthrodesis or TMJ replacement, are reserved for refractory cases [49].

Extra-articular Fractures

A transverse or short oblique fracture line defines extra-articular fractures of the first metacarpal base, resulting in two separate fragments. The oblique posteromedial ligament anchors the proximal fragment to the trapezium and counteracts the dislocating force of the abductor pollicis longus [27]. Conversely, the medial thenar muscles cause displacement and adduction of the distal fragment, potentially narrowing the first web space if untreated. Rarely, an opposite abduction deformity may occur [50].

Palmar comminution can also occur in these fractures (Winterstein fracture). The resultant deformity often involves apex dorsal angulation and adduction due to the combined action of the abductor pollicis longus with an extension of the proximal fragment occurring concurrently with flexion of the distal fragment by the intrinsic thenar muscles [51]. While angular deformity of up to 30° may be tolerated due to TMJ compensation [8], greater angulation warrants manipulation to avoid excessive metacarpophalangeal (MCP) joint hyperextension.

Closed reduction typically involves longitudinal traction, dorsal pressure, gentle pronation, and thumb extension. Stable fractures may be treated conservatively with casting, which does not necessarily need to include the distal phalanx. Unstable oblique fractures may require stabilization using intermetacarpal CRPP or ORIF with one or more lag screws [40]. For unstable transverse fractures, locking plates through a radiopalmar (Wagner) or dorsal approach is preferred, with T-plates being more commonly used. The use of double-row locking plates is linked to a decrease in secondary displacement [52].

Outcome and complications

The prognosis of thumb base fractures depends on several factors. Malreductions can result in short-term complications such as stiffness or instability and long-term issues like radiographic arthritis. Favorable outcomes in these fractures are associated with acute intervention and extra-articular fractures. Conversely, negative prognostic factors include Bennett and Rolando fractures, severely comminuted patterns, and delayed treatment, which may increase the risk of complications and poorer functional outcomes.

Complications of thumb base fractures include post-traumatic arthritis, although its exact incidence remains unclear. Key risk factors contributing to its development are highly comminuted intra-articular fractures, significant articular step-offs, multiple small fragments, and malunion. These factors can compromise joint congruity and stability, leading to long-term degenerative changes.

## Conclusions

The management of fractures at the base of the thumb metacarpal remains a complex yet vital aspect of hand surgery, given the thumb’s critical role in overall hand functionality. While stable extra-articular fractures often respond well to conservative approaches, unstable intra-articular fractures such as Bennett, Rolando, and comminuted patterns require surgical intervention to restore anatomical alignment and preserve joint congruity. Advancements in surgical techniques, including percutaneous pinning, ORIP, and arthroscopic approaches, have significantly improved outcomes for these challenging injuries. However, treatment must be tailored to the fracture pattern, degree of displacement, and individual patient factors to optimize functionality and minimize long-term complications such as arthritis and joint instability. Further research is needed to refine surgical strategies and establish consensus guidelines for managing these diverse fractures.

## References

[1] Brown MT, Rust PA (2020). Fractures of the thumb metacarpal base. Injury.

[2] Stanton JS, Dias JJ, Burke FD (2007). Fractures of the tubular bones of the hand. J Hand Surg Eur Vol.

[3] McGuigan FX, Culp RW (2002). Surgical treatment of intra-articular fractures of the trapezium. J Hand Surg Am.

[4] Garcia-Elias M, Henríquez-Lluch A, Rossignani P, Fernandez de Retana P, Orovio de Elízaga J (1993). Bennett's fracture combined with fracture of the trapezium. A report of three cases. J Hand Surg Br.

[5] Bennett EH (1882). Fractures of the metacarpal bones. Dublin J Med Sci.

[6] Rolando S (1996). Fracture of the base of the first metacarpal and a variation that has not yet been described. Clin Orthop Relat Res.

[7] Hove LM (1993). Fractures of the hand. Distribution and relative incidence. Scand J Plast Reconstr Surg Hand Surg.

[8] Stern PJ (2005). Fractures of the metacarpals and phalanges. Green’s Operative Hand Surgery.

[9] Edwards GA, Giddins GE (2017). Management of Bennett's fractures: a review of treatment outcomes. J Hand Surg Eur Vol.

[10] Malisorn S (2024). The current concept and evidence-based practice in the base of the first metacarpal bone fracture. Cureus.

[11] Kjaer-Petersen K, Langhoff O, Andersen K (1990). Bennett's fracture. J Hand Surg Br.

[12] Greeven AP, Alta TD, Scholtens RE, de Heer P, van der Linden FM (2012). Closed reduction intermetacarpal Kirschner wire fixation in the treatment of unstable fractures of the base of the first metacarpal. Injury.

[13] Foster RJ, Hastings H (1987). Treatment of Bennett, Rolando, and vertical intraarticular trapezial fractures. Clin Orthop Relat Res.

[14] Bettinger PC, Linscheid RL, Berger RA, Cooney WP 3rd, An KN (1999). An anatomic study of the stabilizing ligaments of the trapezium and trapeziometacarpal joint. J Hand Surg Am.

[15] McCann MR, Rust PA, Wallace R (2018). The stabilising effect of the anterior oblique ligament to prevent directional subluxation at the trapeziometacarpal joint of the thumb: a biomechanical cadaveric study. Arch Bone Jt Surg.

[16] Edmunds JO (2006). Traumatic dislocations and instability of the trapeziometacarpal joint of the thumb. Hand Clin.

[17] Foucher G (1982). Injuries of the trapezo-metacarpal joint. (Article in French). Ann Chir Main.

[18] (2024). Base of thumb fractures. https://www.orthobullets.com/hand/6036/base-of-thumb-fractures.

[19] Gedda KO (1954). Studies on Bennett's fracture; anatomy, roentgenology, and therapy. Acta Chir Scand Suppl.

[20] Lutz M, Sailer R, Zimmermann R, Gabl M, Ulmer H, Pechlaner S (2003). Closed reduction transarticular Kirschner wire fixation versus open reduction internal fixation in the treatment of Bennett's fracture dislocation. J Hand Surg Br.

[21] Robert P (1936). Bulletins and memories of the society of radiology. (Article in French). Medicate de France.

[22] Lewis S (1988). New angles on the radiographic examination of the hand-I. Radiogr Today.

[23] Billing L, Gedda KO (1952). Roentgen examination of Bennett's fracture. Acta Radiol (Stockh).

[24] (2024). Adult trauma. https://surgeryreference.aofoundation.org/orthopedic-trauma/adult-trauma.

[25] Capo JT, Kinchelow T, Orillaza NS, Rossy W (2009). Accuracy of fluoroscopy in closed reduction and percutaneous fixation of simulated Bennett's fracture. J Hand Surg Am.

[26] Kapandji A, Moatti E, Raab C (1980). Specific radiography of the trapezo-metacarpal joint and its technique. (Article in French). Ann Chir.

[27] Liverneaux PA, Ichihara S, Hendriks S, Facca S, Bodin F (2015). Fractures and dislocation of the base of the thumb metacarpal. J Hand Surg Eur Vol.

[28] Cullen JP, Parentis MA, Chinchilli VM, Pellegrini VD Jr (1997). Simulated Bennett fracture treated with closed reduction and percutaneous pinning. A biomechanical analysis of residual incongruity of the joint. J Bone Joint Surg Am.

[29] Oosterbos CJ, de Boer HH (1995). Nonoperative treatment of Bennett's fracture: a 13-year follow-up. J Orthop Trauma.

[30] Lambotte A (1913). Operative Surgery for Fractures. (Book in French). Internet]. Paris : Masson & Cie.

[31] Wagner CJ (1950). Method of treatment of Bennett's fracture dislocation. Am J Surg.

[32] Bennani A, Zizah S, Benabid M (2012). The intermetacarpal double pinning in the surgical treatment of Bennett fracture (report of 24 cases). (Article in French). Chir Main.

[33] Leclère FM, Jenzer A, Hüsler R, Kiermeir D, Bignion D, Unglaub F, Vögelin E (2012). 7-year follow-up after open reduction and internal screw fixation in Bennett fractures. Arch Orthop Trauma Surg.

[34] Zemirline A, Lebailly F, Taleb C, Facca S, Liverneaux P (2014). Arthroscopic assisted percutaneous screw fixation of Bennett's fracture. Hand Surg.

[35] Iselin M, Blanguernon S, Benoist D (1956). First metacarpal base fractures. (Article in French). Mem Acad Chir (Paris).

[36] Adi M, Miyamoto H, Taleb C, Zemirline A, Gouzou S, Facca S, Liverneaux P (2014). Percutaneous fixation of first metacarpal base fractures using locked K-wires: a series of 14 cases. Tech Hand Up Extrem Surg.

[37] Meyer C, Hartmann B, Böhringer G, Horas U, Schnettler R (2003). Minimal invasive cannulated screw osteosynthesis of Bennett's fractures. (Article in German). Zentralbl Chir.

[38] Moberg E, Gedda KO (1952). Surgical therapy of Bennett's fracture. Nord Med.

[39] Eaton RG, Lane LB, Littler JW, Keyser JJ (1984). Ligament reconstruction for the painful thumb carpometacarpal joint: a long-term assessment. J Hand Surg Am.

[40] van Niekerk JL, Ouwens R (1989). Fractures of the base of the first metacarpal bone: results of surgical treatment. Injury.

[41] Rolando S, Meals RA (2006). Fracture of the base of the first metacarpal and a variation that has not yet been described. Clin Orthop Relat Res.

[42] Vichard P, Tropet Y, Nicolet F (1982). Longitudinal pinning of fractures of the base of the first metacarpal. Ann Chir Main.

[43] van Leeuwen WF, van Hoorn BT, Chen N, Ring D (2016). Kirschner wire pin site infection in hand and wrist fractures: incidence rate and risk factors. J Hand Surg Eur Vol.

[44] Gelberman RH, Vance RM, Zakaib GS (1979). Fractures at the base of the thumb: treatment with oblique traction. J Bone Joint Surg Am.

[45] Florek J, Georgiew F, Petrovych O, Florek P, Janowiec S (2024). Non-traditional surgical treatment of a Rolando fracture. Cureus.

[46] Buchler U, McCollam SM, Oppikofer C (1991). Comminuted fractures of the basilar joint of the thumb: combined treatment by external fixation, limited internal fixation, and bone grafting. J Hand Surg Am.

[47] Culp RW, Johnson JW (2010). Arthroscopically assisted percutaneous fixation of Bennett fractures. J Hand Surg Am.

[48] Pomares G, Strugarek-Lecoanet C, Dap F, Dautel G (2016). Bennett fracture: arthroscopically assisted percutaneous screw fixation versus open surgery: functional and radiological outcomes. Orthop Traumatol Surg Res.

[49] Wagner CJ (1951). Transarticular fixation of fracture-dislocations of the first metacarpal-carpal joint. West J Surg Obstet Gynecol.

[50] Kapandji IA (1983). A technique for crossed pinning on non articular fractures of the base of the first metacarpal. (Article in French). Ann Chir Main.

[51] Carlsen BT, Moran SL (2009). Thumb trauma: Bennett fractures, Rolando fractures, and ulnar collateral ligament injuries. J Hand Surg Am.

[52] Diaconu M, Facca S, Gouzou S, Liverneaux P (2011). Locking plates for fixation of extra-articular fractures of the first metacarpal base: a series of 15 cases. Chir Main.

